# Research on Prophages in Aquaculture from the Perspective of Paradigm Borrowing: Advances in Pathogen Virulence, Antimicrobial Resistance, and Environmental Regulation

**DOI:** 10.3390/microorganisms14091904

**Published:** 2026-08-28

**Authors:** Ziqiao Zhao, Yixin Li, Yunhan Li, Chengshuo Shen, Youyu Liu, Peng Zhang

**Affiliations:** Yantai Institute of China Agricultural University, China Agricultural University, Yantai 264670, China; zhaoziqiao777@163.com (Z.Z.); m17763309060@163.com (Y.L.); 2024505430134@cau.edu.cn (Y.L.); 15820069307@163.com (C.S.); liuyouyu1204@163.com (Y.L.)

**Keywords:** prophages, aquatic bacterial pathogens, virulence factors, antimicrobial resistance genes, competitive advantage, environmental regulation

## Abstract

Prophages are widespread in bacterial genomes and can influence pathogen evolution by modulating virulence, antimicrobial resistance, environmental adaptation, and competitive fitness. However, prophage research in aquaculture pathogens remains limited, with abundant genomic predictions but scarce functional validation, numerous cross-sectional surveys but little longitudinal tracking, and extensive phenomenological observations but insufficient mechanistic investigation. This review synthesizes current knowledge of prophages in aquatic bacterial pathogens, focusing on their roles in virulence, antimicrobial resistance, and competitive advantage, while drawing on mechanistic paradigms established in human and animal pathogens. We further propose a pathogen-centered “molecular bridge” framework in which environmental perturbations alter bacterial physiological states, are interpreted through prophage regulatory mechanisms, and ultimately translate into changes in pathogen phenotypes, fitness, and pathogenic potential. Particular attention is given to multi-signal interactions, induction thresholds, and the context-dependent fitness consequences of prophage induction in dynamic aquaculture environments. Finally, we propose future priorities encompassing systematic prophage resource construction, functional validation, multi-stressor experiments, and longitudinal monitoring. By adopting a paradigm-borrowing review approach, this review aims to provide a transferable roadmap for prophage research in aquaculture, to facilitate translational applications, and to offer theoretical support for precision disease control in aquaculture.

## 1. Introduction

Phages exhibit diverse infection strategies [1,2,3]. Virulent phages are classically characterized by a predominantly lytic infection strategy, in which productive infection typically involves phage replication, host-cell lysis, and release of progeny virions [4]. Temperate phages, by contrast, can alternate between the lytic and lysogenic pathways [5]. Although virulent and temperate lifestyles represent the two classical and best-characterized categories, this binary classification does not capture the full diversity of phage-host interactions [6]. Since the present review focuses on prophage biology, the following discussion primarily concerns temperate phages—namely, phages capable of establishing stable prophage states within bacterial hosts.

During the lysogenic cycle, temperate phages integrate their genomes into the bacterial chromosome as prophages and replicate in concert with the host genome. Prophages can also excise from the host genome through spontaneous excision or in response to external stress-inducing signals, thereby triggering the lytic cycle and causing host lysis [4,5,7]. The term “prophage” refers to a temperate phage genome that is integrated into the bacterial chromosome or maintained as an extrachromosomal plasmid within the bacterial cell; bacteria that carry prophages are termed lysogens, and this state is referred to as lysogeny [8].

Lysogenic conversion refers to a potential phenotypic alteration in a lysogenic bacterium arising from the expression or regulatory effects of prophage-associated genes [8]. Importantly, lysogeny does not necessarily lead to lysogenic conversion, as prophage genes can remain transcriptionally silent; their expression may depend on the bacterial genetic background, host regulatory networks, and specific environmental cues. Upon functional expression, prophage-associated genes can modulate diverse bacterial traits, including virulence, antimicrobial resistance, metabolism, stress tolerance, and interactions with eukaryotic hosts or competing microorganisms [9,10,11,12]. Lysogenic conversion thus constitutes one important yet context-dependent mechanism by which prophages shape bacterial phenotypes. Furthermore, prophages can be classified as functional prophages or defective prophages. Functional prophages can resume the lytic cycle upon induction and produce infectious progeny particles. Defective prophages, by contrast, have lost the ability to produce progeny due to genomic deletions, insertional disruptions, or point mutations, yet may still retain functional genes (such as toxin genes) that contribute to the host phenotype [13,14]. A complex “arms race” exists between prophages and bacterial defense systems; prophages can encode anti-CRISPR (Acr) proteins, such as AcrIIA5 and AcrIIA23, that inhibit the activity of CRISPR-Cas systems, whereas bacteria can acquire spacers from their own prophages to establish self-targeting immunity [15,16,17,18,19]. Moreover, prophages are frequently co-localized with defense gene clusters, including restriction-modification systems and toxin-antitoxin systems, within “defense islands” [20,21]. In summary, prophages have exerted a considerable influence on bacterial adaptation and evolution, and sustained and in-depth investigation is warranted.

With the explosive growth of bacterial whole-genome sequencing data, prophages have been found to reside in nearly all bacterial genomes, and they are also ubiquitous in aquatic bacterial pathogens [22,23,24,25,26,27,28,29]. Current progress in prophage research on aquatic pathogens is summarized in Table 1. Therefore, scientifically dissecting the distribution patterns, biological functions and ecological risks of prophages in aquaculture pathogens is of considerable theoretical and practical importance for informing phage therapy and disease control in aquaculture. However, the current research landscape in this field is characterized by a notable pattern of “three abundances and three scarcities”—namely, abundant genomic predictions but scarce functional validation, abundant cross-sectional surveys but scarce longitudinal tracking, and abundant phenomenological descriptions but scarce mechanistic dissection. This state of research stands in marked contrast to the mature paradigms that have been established in studies of human and animal pathogens.

These three gaps are closely interconnected and cannot simply be filled by accumulating more prophage sequences or descriptive surveys. Genomic predictions require functional experiments to establish whether prophage-encoded elements indeed alter pathogen phenotypes; cross-sectional observations require longitudinal tracking approaches to determine how prophage status changes in response to environmental conditions and disease progression; and phenomenological correlations require mechanistic frameworks that can link environmental signals, prophage regulation, and pathogen responses. Given that mechanistic evidence available for aquaculture pathogens remains limited, the well-established concepts and experimental paradigms from human and animal pathogen research provide a valuable starting point for formulating testable scientific questions.

Based on the above considerations, this review aims to achieve the following objectives, (1) to examine the existing evidence on prophages in aquaculture pathogens across the three dimensions of virulence, antimicrobial resistance and competitive advantage, thereby clarifying the current knowledge base and its boundaries, (2) to distil and draw upon mature experimental paradigms and theoretical frameworks from human and animal pathogen research, and to explore their applicability and translational potential in aquaculture contexts, (3) to diagnose the gaps and pinpoint the bottlenecks that impede the transition from descriptive to causal research in aquaculture, and (4) to construct a forward-looking pathway by proposing solutions that encompass the building of genomic resources, the establishment of functional validation systems, longitudinal dynamic monitoring and the integration of ecological theory. By adopting a narrative approach that seeks to “drive the field forward through paradigm borrowing”, this review aims to provide a transferable roadmap for prophage research in aquaculture and to offer theoretical support for precision disease control and early-warning systems for antimicrobial resistance risk in aquaculture.

## 2. Prophage-Conferred Virulence, Antimicrobial Resistance, and Competitive Advantage in Aquatic Bacterial Pathogens

**Figure 1:**
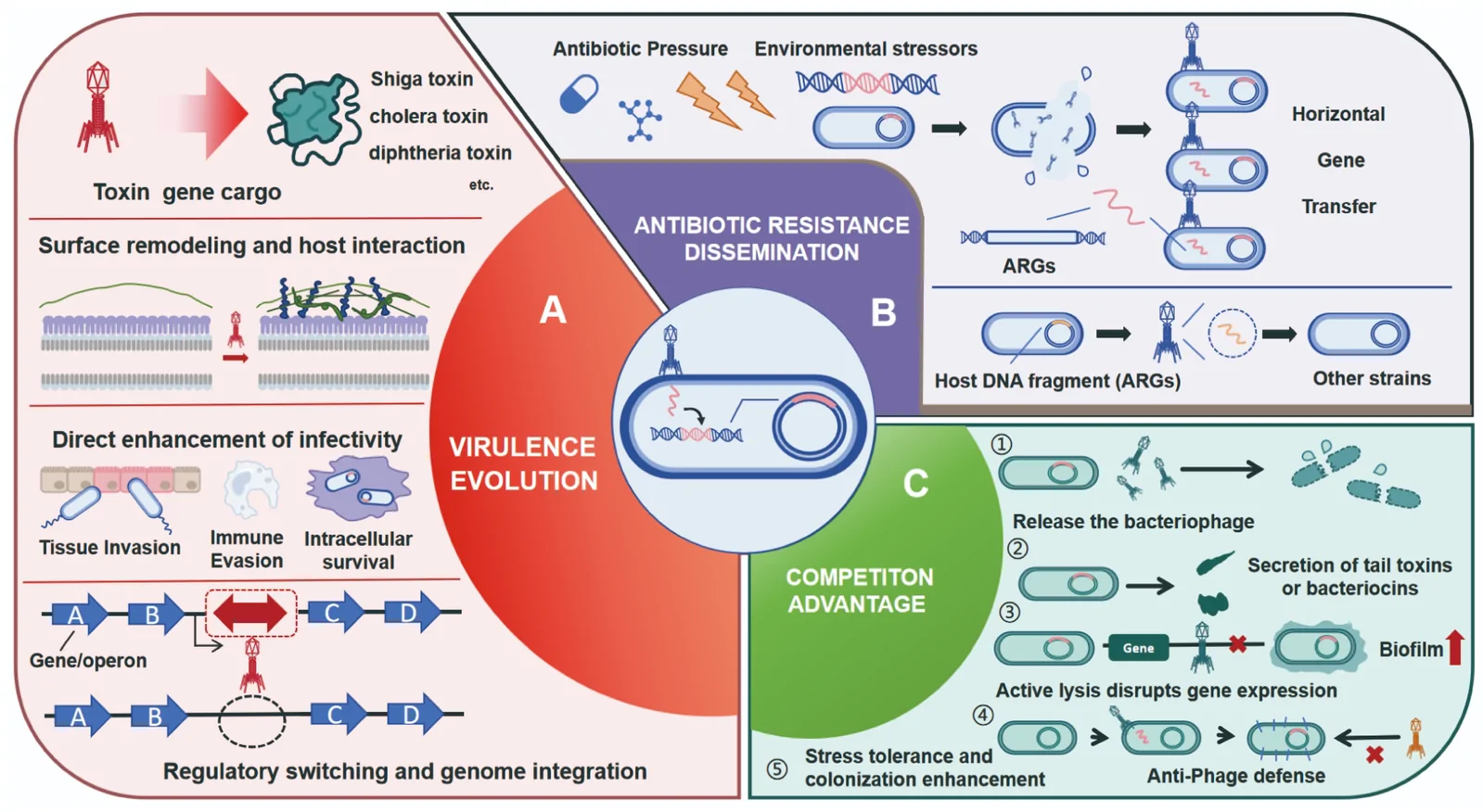
Prophage-conferred virulence, antimicrobial resistance, and competitive advantage in bacterial pathogens. Prophages are involved in the dissemination and regulation of virulence factors (**A**), are closely associated with the spread of antimicrobial resistance genes (**B**) and enhance host fitness through diverse competitive mechanisms (**C**).

Recent studies have indicated that prophages are not only involved in the dissemination and regulation of virulence factors but are also closely linked to the spread of antimicrobial resistance genes and can enhance the ecological fitness of host bacteria through various competitive mechanisms (Figure 1). In the following sections, we discuss research progress on prophages in aquaculture across these three dimensions—virulence, antimicrobial resistance, and competitive advantage—and draw upon research paradigms from other fields to offer methodological and theoretical insights for aquaculture.

### 2.1. Prophage-Mediated Virulence Factors in Aquaculture

The phenomenon by which prophages confer virulence factors to their bacterial hosts through lysogenic conversion was first discovered in human pathogens. In pathogens of humans and other animals, prophages influence bacterial virulence through multiple mechanisms. One mechanism is the secretion of phage-encoded toxins, as exemplified by the CTXφ prophage in *Vibrio cholerae* encoding cholera toxin, prophages in Shiga toxin-producing *Escherichia coli* (STEC) encoding Shiga toxin, and the prophage in *Corynebacterium diphtheriae* encoding diphtheria toxin [9,35,36]. A second mechanism involves modulation of the bacterial envelope structure, which affects virulence by altering bacterial surface components. For instance, in *Serratia marcescens*, a temperature-sensitive phage has been found to alter virulence in insect hosts; genetic and epigenetic analyses have suggested that this change is attributable to modifications in the outer cell wall structure [37]. In *Ralstonia solanacearum*, phage ϕRSS1 alters surface properties (e.g., hydrophobicity) and increases production of virulence factors such as exopolysaccharide (EPS), thereby contributing to enhanced virulence [38]. A third mechanism is the direct mediation of bacterial infectivity by prophages. For example, in *Salmonella Typhimurium*, the Gifsy-2 prophage provides genes encoding *gtgE* and *sodCI*, which contribute to enhanced virulence [39]. A fourth mechanism involves the modulation of bacterial regulatory circuits. Prophages can integrate into key genes or operons within the bacterial chromosome and act as switches to regulate gene expression through genomic excision [40]. For instance, *Listeria monocytogenes* carries a prophage within its *comK* gene, which remains in an inactive state [41]. Upon infection of macrophages, this prophage switches to an active state that promotes *comK* expression, thereby facilitating intracellular bacterial growth and contributing to virulence [41].

In recent years, with the rapid accumulation of whole-genome sequencing data for aquatic bacterial pathogens, researchers have begun to investigate the role of prophages in the virulence evolution of aquaculture pathogens. The understanding of prophage-mediated virulence regulation in aquatic pathogens remains considerably less advanced than that in pathogens of higher animals. Nevertheless, it is possible to draw upon established research paradigms from higher animals to inform investigations in aquaculture settings.

Among the various aspects of prophage research in aquaculture, reports of prophage-carried virulence genes are more frequently documented than those on other topics. Nevertheless, most relevant findings are still based on computational predictions, although several Level 2 empirical studies have been reported (Table 1). Within the genus *Vibrio*, for example, six out of nine highly virulent strains of *Vibrio anguillarum* possess unique accessory genomes that contain pathogenicity genomic islands, prophage-like elements, and gene clusters involved in polysaccharide biosynthesis, whereas strains of moderate and low virulence display greater genomic homogeneity [42]. This finding suggests that prophage-like elements may be closely linked to the virulence evolution of *V. anguillarum*. A T6SS virulence gene was detected in a prophage-like element of *Vibrio parahaemolyticus* strain A1, and capsular polysaccharide virulence genes (*wbfV*/*wcvB* and *wecA*) were identified in a defective prophage of *Vibrio alginolyticus* strain B14 [24]. Furthermore, prophages phi354 and phiGAN1709 encode the Flp (fimbrial low-molecular-weight protein) family of type IV pili, which in *Vibrio vulnificus* promote host cell and tissue invasion, survival in blood, and resistance to serum bactericidal activity, thereby markedly enhancing bacterial virulence [31,43]. pAOD131 and pGAN1807 encode an ATP-dependent Clp protease proteolytic subunit, prophage phi354 encodes VapD (virulence-associated protein D), and prophages that are widely distributed in *Vibrio harveyi* encode PadR. Moreover, PadR has been identified as the most prevalent virulence factor among prophages in *V. harveyi* [31]. VapD is a thermostable ribonuclease whose elevated expression is associated with biofilm formation in virulent strains of *Xylella fastidiosa* [44]. PadR can regulate genes involved in detoxification, virulence, and multidrug resistance [45]. Additionally, a homologous protein VpaChn25_0724 from a Mu-like prophage in *Vibrio parahaemolyticus* strain CHN25 affects host cell motility, biofilm formation, and cytotoxicity [46].

Moreover, the integration of a prophage into the CRISPR locus can enable *Streptococcus pyogenes* to evade the antiviral immune response [16]. In the genus *Flavobacterium*, the identification of prophage 6H across five genomes of *Flavobacterium psychrophilum* corroborates a recent finding that this prophage exhibits widespread and global distribution among *F. psychrophilum* isolates. Furthermore, prophage 6H has been linked to potential virulence factors, and is inferred to encode a LuxR-family transcriptional regulator and proteins similar to IbrA and IbrB [30]. In the genus *Aeromonas*, several *Aeromonas hydrophila* strains harbor Shiga toxin-converting prophages, such as 933W and Stx2. An intact enterobacterial prophage 933W was detected in strain Ah-10 (other ST group), whereas Stx2 prophage vB_EcoP_24B was identified in strains 2JBN101, D4, and HZAUAH (ST-251). Notably, all strains carrying Shiga toxin prophages were isolated from fish [26].

Re-examining the available evidence in aquaculture through the lens of the four categories of mechanisms outlined above reveals that, although a large number of potential virulence-associated prophage elements have been identified in aquatic pathogens in recent years with the accumulation of whole-genome sequencing data, the vast majority of studies remain at the stage of sequence association or functional conjecture (Table 1 and Table 2). In terms of the level of evidence, these studies primarily address the question of “which prophage genes may be associated with virulence,” but have not yet answered the questions of “by what mechanisms, under what environmental or infection conditions, and to what extent these genes alter pathogen virulence.” Consequently, what is truly worth transferring from human and animal pathogen research to the aquaculture setting is not the specific fact that “phage X carries gene Y,” but rather the well-established causal research paradigms that underpin such findings.

### 2.2. Prophages and Dissemination of Antimicrobial Resistance Genes

In other fields, the mechanisms by which prophages mediate the dissemination of antimicrobial resistance genes have been extensively documented, and several representative research paradigms have emerged. First, generalized transduction represents a classical route for the horizontal transfer of resistance genes across cells. In *Acinetobacter baumannii*, prophages can randomly package host genomic DNA fragments and efficiently transfer chromosomal antibiotic resistance genes to other strains without requiring direct cell-to-cell contact [47]. These resistance-conferring extracellular DNA fragments are encapsulated within phage coat proteins, thereby being protected from degradation by DNase and detergents [47]. Second, antibiotic exposure can induce prophage-mediated gene transfer. For example, in *Salmonella Typhimurium*, exposure to carbadox induces the release of phage particles that can co-transfer virulence genes and resistance genes to susceptible recipient strains [48]. Notably, non-antibiotic environmental pollutants can exert similar inducing effects. Non-antibiotic pollutants such as triclosan and silver nanoparticles can trigger prophage induction through the elicitation of excessive oxidative stress, thereby promoting the horizontal dissemination of resistance genes [49]. Collectively, the mature research paradigms in higher animals have, through transduction experiments, conjugation assays, and animal models, definitively demonstrated the critical roles of generalized transduction and antibiotic-induced transduction in the horizontal dissemination of resistance genes across diverse pathogenic bacteria. In aquaculture settings, it is plausible that similar dissemination mechanisms exist, and these warrant systematic investigation.

In aquaculture, however, studies directly establishing functional causal links between prophages and antimicrobial resistance in well-defined aquatic bacterial pathogens remain scarce. Current evidence is derived primarily from community-level investigations of aquaculture-associated microbiomes, rather than experimentally validated pathogen-prophage systems. It must be emphasized, however, that these studies have only established genome-scale statistical associations, and do not constitute direct functional evidence for ARG dissemination. In crab aquaculture ponds and ditches, co-occurrence network analysis (Spearman r > 0.7, *p* < 0.01) identified statistical associations between prophages and multiple ARG subtypes (*rsmA*, *OXA-21*, *THIN-B*, *lnuF*) [50]. These observations suggest only correlative links between prophages and ARGs, rather than providing direct evidence of physical linkage, phage-mediated packaging, transfer direction, successful transduction, or causal relationships. Notably, shifts in gene abundance derived from metagenomic data cannot equate to gene expression, extracellular release, predicted mobility, or experimentally validated gene transfer. Further investigations suggest that antibiotic use may promote the emergence and spread of ARGs, a process that might also be intimately linked to prophage activity. For example, in a study examining prophylactic application of sarafloxacin hydrochloride (SAR) in Pacific white shrimp (*Litopenaeus vannamei*), SAR exposure upregulated key SOS-response regulators (*recA*, *recX*, and *lexA*), indicating activation of the SOS response [51]. This activation was correlated with prophage induction, as reflected by a reduction in prophage abundance, and was accompanied by changes in the abundance of ARGs and virulence factors [51]. However, it should be noted that this study was conducted solely through metagenomic sequencing and bioinformatic analyses; therefore, these observations represent correlative evidence rather than direct experimental validation of causal relationships.

Beyond aquaculture, researchers have employed the deep-learning classifier Cherry, which integrates compositional features, coverage covariation, and CRISPR spacer matching to establish phage–host associations and assign phage–host relationships at genomic resolution [52]. Concurrently, analyses by CW revealed a substantial overlap between hosts of pathogenic, antibiotic-resistant bacteria (PARB) and those of phages. Hosts harboring pathogenic phages were found to be abundant and highly active in plant and sediment compartments, thereby contributing to the abundance and expression levels of PARB [52]. Collectively, these patterns suggest the existence of an active phage-mediated pathway that substantially increases the risk of horizontal transfer of antibiotic resistance genes (ARGs) via transduction both within and among pathogenic bacterial populations [52]. This study also provides indirect evidence that prophages can mediate ARG transfer in aquatic environments.

In summary, prophages are not only one of the drivers of antimicrobial resistance gene dissemination in aquaculture settings, but stressful conditions caused by antibiotic and non-antibiotic pollutants may also further promote prophage induction and the spread of ARGs, for instance through activation of the SOS response. Although research specifically addressing the relationship between prophages and antimicrobial resistance genes in aquaculture remains limited, the continued emergence and spread of resistant strains, together with the growing challenge of antimicrobial resistance, underscores the need for in-depth investigations into the precise roles of prophages in facilitating the dissemination of resistance genes.

### 2.3. Prophage-Conferred Competitive Advantage

Prophages also encode a variety of competitive exclusion mechanisms that confer fitness advantages on their bacterial hosts. Given the notable knowledge gaps but considerable investigative potential of prophage research in aquaculture, it is reasonable to draw on classical studies from human and animal pathogens, leveraging cross-species generalizable mechanisms and principles to inform methodological approaches in the aquatic field [36].

Bacteria can utilize prophages to kill sensitive competitors or secrete antimicrobial elements that directly eliminate niche rivals. For example, *Pseudomonas aeruginosa* can gain substantial fitness advantages in mixed infections through prophage-mediated interbacterial competition [53]. In addition, prophage-encoded tailocins in *Pseudomonas* spp. can be used to kill coexisting competitor strains, and tailocin variants carried by different strains can target variable polysaccharides in the outer membranes of competitors [54]. Similarly, in *Marinomonas mediterranea*, prophages that degenerate into defective prophages can promote the synthesis of R-type bacteriocins, which constitute an important mechanism for competitive colonization in habitats such as marine plant surfaces [55]. Notably, *Marinomonas mediterranea*, although not a pathogen, provides a reference paradigm for competitive colonization by pathogens through its prophage-mediated bacteriocin mechanism. Moreover, spontaneous prophage induction in a small subset of cells within a lysogenic population can enhance the competitive fitness of the entire lysogenic population [56,57].

Phages can also drive rapid adaptation through active lysogeny, whereby an integrated prophage inserts into a functional gene and disrupts its expression, thereby conferring fitness benefits [58]. Studies suggest that this process can promote rapid, parallel adaptive changes, for instance, in *P. aeruginosa*, such recombination events repeatedly disrupt genes encoding global regulators, leading to elevated cyclic di-GMP levels and increased biofilm production [58]. The adaptive effects mediated by prophages may have broad implications, as even transient members of microbial communities can thereby influence evolutionary trajectories [58].

Bacteria can also enhance their resistance to phage attack through prophage-encoded anti-phage defense systems, such as superinfection exclusion (SIE) mechanisms. Prophages encode multiple anti-phage defense systems, including innate-like immune mechanisms such as restriction-modification systems and SIE proteins, as well as adaptive immune systems like CRISPR-Cas [59]. Lysogens carrying these defense systems have a marked selective advantage in phage-rich environments [60,61]. For example, structural proteins encoded by the filamentous prophage of *P. aeruginosa* can mediate superinfection exclusion by interfering with type IV pilus function, effectively preventing secondary infection by homologous phages [62]. In aquaculture environments, which are characterized by high phage densities, the competitive advantage conferred by SIE on prophage-carrying strains may be particularly important; however, direct studies of SIE mechanisms in aquatic pathogens are currently lacking.

Furthermore, prophages can indirectly enhance competitive fitness by increasing tolerance to environmental stressors and improving colonization capacity. In avian pathogenic *Escherichia coli* (APEC), the prophage phiv142-3 contributes to bacterial survival under adverse conditions and promotes colonization—strains lacking this prophage exhibit reduced colony size, elongated cell morphology, and significantly diminished adhesion and serum resistance [63].

In summary, the well-established mechanisms by which prophages confer competitive advantages in higher animals have been clearly demonstrated through gene knockout, competition assays, and animal models. These mechanisms include the killing of sensitive competitors, synthesis of tailocins/bacteriocins, disruption of regulatory genes via active lysogeny to drive rapid adaptation, enhancement of tolerance to environmental stressors and colonization capacity, and superinfection exclusion. In aquaculture, however, research in this area remains notably scarce. It is therefore imperative to draw upon the mechanisms and experimental strategies established in the non-aquatic studies discussed above, so as to advance research in this field.

## 3. Conceptual Framework: Prophages as a Molecular Bridge Between Environmental Signals and Pathogen Phenotypic Outcomes

**Figure 2:**
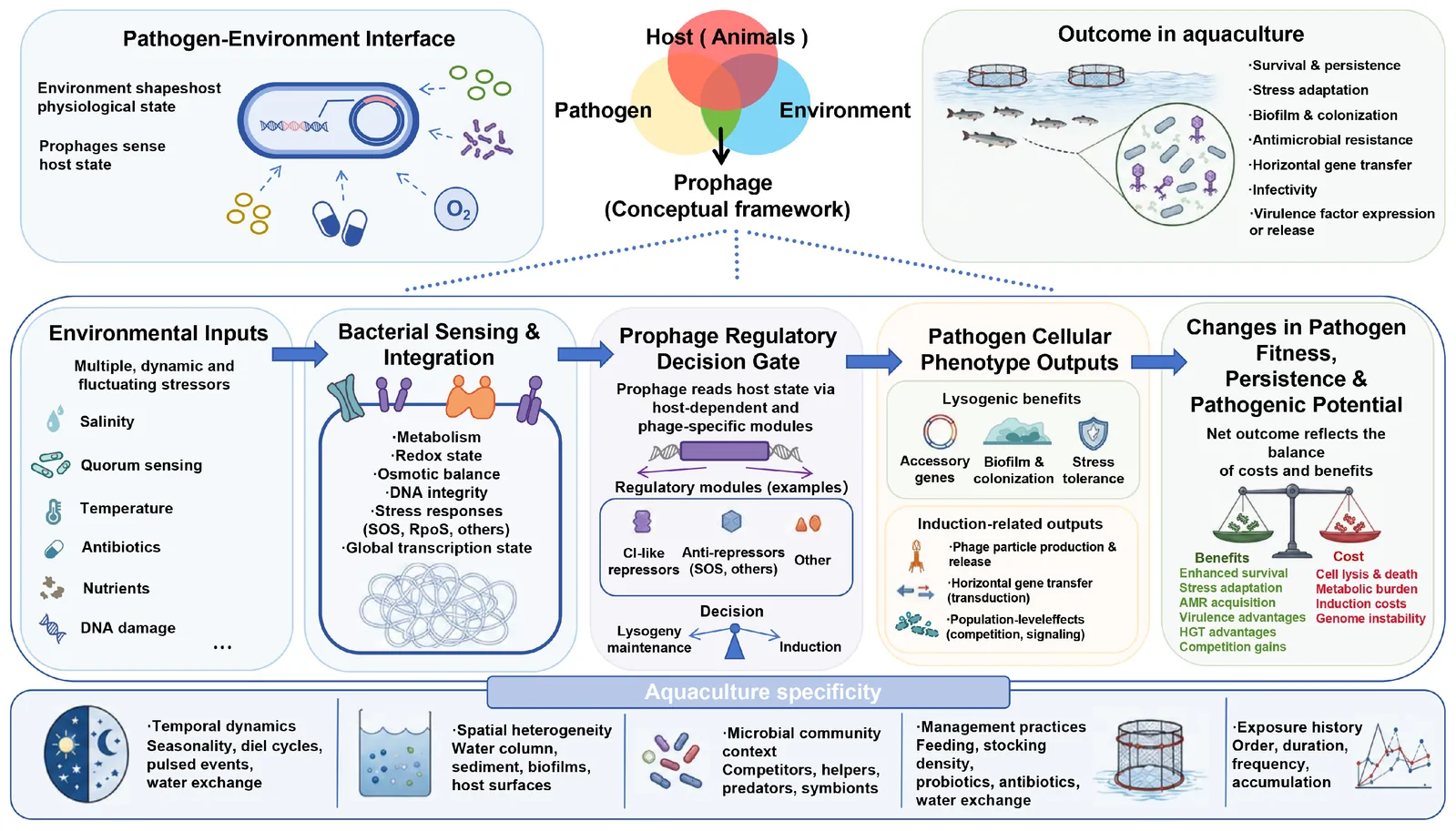
Proposed conceptual framework: prophage effects on pathogen phenotypes in aquaculture. This framework synthesizes current evidence and testable hypotheses, rather than confirmed mechanisms. The model links five steps—environmental inputs, bacterial sensing, prophage regulatory decision gates, pathogen phenotypic outputs, and fitness consequences—and emphasizes that the net outcome depends on signal integration, temporal dynamics, and population-level heterogeneity.

In the classical paradigms of immunology and infectious diseases, pathogens, hosts, and the environment interact dynamically, collectively determining disease occurrence and outcome. Prophages, temperate phages integrated into bacterial genomes, are situated precisely at the core of the pathogen–environment interface (Figure 2). Understanding the importance of the interplay between prophages and their environmental contexts and host pathogens is therefore critical for elucidating the ecological adaptive evolution of pathogens in aquaculture settings, as well as for developing environment-based strategies for disease prevention and control. However, given the scarcity of research in this area within aquaculture, it is reasonable to draw upon classical studies in human and animal pathogens, leveraging cross-species generalizable mechanisms and principles to inform methodological approaches in the aquatic field [36].

To move the “molecular bridge” beyond a purely metaphorical description, we operationalize it here as a conceptual model comprising five sequential yet reciprocally interacting steps: (1) environmental input; (2) bacterial sensing and integration of environmental signals; (3) prophage regulatory decision gates; (4) phenotypic output of the pathogen cell; and (5) changes in pathogen fitness, persistence, and pathogenic potential. In this model, environmental factors do not directly determine the fate of the prophage; rather, they first alter the physiological and regulatory state of the host bacterium, and these changes are subsequently interpreted by the prophage-specific sensing and regulatory modules, which in turn modulate the probability of lysogenic maintenance versus lytic induction. The ultimate ecological outcome is shaped not only by whether prophage induction occurs, but also by the proportion of induced cells, the intensity and duration of environmental stress, the combination and temporal sequence of signals, and the composition of the surrounding microbial community.

### 3.1. Environmental Inputs: From External Perturbations to the Integrated Physiological State of the Host Bacterium

In other fields, the host signals currently implicated in modulating prophage states primarily include DNA damage and the RecA–LexA-dependent SOS response [64], quorum-sensing signals [65], oxidative stress [66], osmotic changes [67], nutrient limitation [68], metabolic status, and host-derived antimicrobial peptides and immune-related factors [69]. However, these signals do not act as independent, simple inputs. For example, nutrient conditions can alter bacterial growth rates and metabolic activity [70]; oxidative stress may simultaneously cause protein damage, membrane injury, and DNA damage [71]; and changes in cell density may reshape global bacterial transcription through quorum-sensing systems [72]. Therefore, it is the integrated physiological state formed within the pathogen cell in response to external perturbations—rather than the external environmental parameters per se—that influences prophage behavior [73]. For instance, the temperate phage VP882 infecting *Vibrio cholerae* can sense host-related quorum-sensing information and adjust its lysogeny–lysis decision accordingly [74]. Furthermore, studies suggest that host quorum-sensing status and metabolic status can jointly influence the fate decisions of temperate phages [75].

This mechanism is beginning to receive direct support in aquatic pathogens as well. In the fish pathogen *Vibrio anguillarum*, high cell density and quorum sensing have been shown to suppress induction of the H20 prophage, while the prophage state can in turn influence host biofilm formation [76]. This suggests that, in aquatic vibrios, cell density, host regulatory networks, and prophage fate are not independent of one another, but may constitute an interconnected regulatory system.

It is worth noting that, in contrast to clinical settings, aquaculture systems are subject to frequent and pronounced fluctuations, including variations in temperature (seasonal shifts, diurnal temperature differences, and water exchange operations), salinity (freshwater input during rainy seasons and evaporative concentration), dissolved oxygen (stocking density, algal photosynthesis and respiration), nutrients (feed input and fecal accumulation), and antibiotic residues (prophylactic and therapeutic use). Such complex fluctuations in environmental factors may exert more intricate effects on the lysogeny–lysis switch of temperate phages.

Different signals may also exhibit additive, synergistic, or antagonistic effects. However, direct experimental evidence for such signal-signal interactions in aquatic pathogens remains scarce, and most current inferences are extrapolated from model bacterial systems. We therefore do not assume that these relationships can be considered established; rather, we treat them as variables to be tested within our framework. For example, if combined exposure to two environmental factors results in a level of prophage induction that is appreciably higher than that predicted from their individual effects, a synergistic interaction could be inferred; conversely, if one form of stress suppresses the induction elicited by another, an antagonistic interaction would be suggested. In addition, the temporal order of signals may also be important. Environmental disturbances in aquaculture systems are often periodic or pulsed in nature; prior exposure to nutrient limitation, hypoxia, or osmotic changes may alter bacterial metabolism and stress responses, thereby influencing subsequent responses to DNA damage or oxidative stress. Collectively, the type, intensity, duration, combination, and exposure history of environmental signals together constitute the “environmental input layer” of our framework.

### 3.2. Prophage Regulatory Decision Gates Determine Whether the Pathogen Crosses the Induction Threshold

The classical lambda phage provides a representative model for understanding how host physiological signals are translated into changes in prophage state. Under stable lysogenic conditions, the CI repressor protein continuously suppresses the expression of lytic genes, thereby maintaining prophage silence [77]. When the host experiences DNA damage and activates the SOS response, the activated RecA promotes the inactivation of CI repression, allowing the lytic transcriptional program to be initiated and driving the transition of the prophage from the lysogenic state to the lytic cycle [77]. In contrast, the CII/CIII regulatory module is primarily involved in the establishment of lysogeny during the initial infection of a temperate phage: CII promotes lysogenic transcription, while CIII enhances this process by increasing the stability of CII [77]. Thus, the classical lambda system illustrates that phage fate is not determined by a single “switch,” but rather by multiple regulatory nodes acting at different stages [78].

However, the “prophage regulatory decision gate” as defined in this Review is not equivalent to the CI–Cro system of lambda phage. Rather, it serves as a conceptual umbrella term for a range of host-dependent and prophage-specific regulatory modules that, across different pathogens, are capable of transducing host physiological states into changes in the probability of prophage induction. In other words, the classical lambda system provides a mechanistic paradigm, rather than a universally applicable molecular model that all prophages in aquatic pathogens necessarily follow.

Studies on polylysogens have further shown that different prophages within the same host genome may respond differently to environmental stimuli. It has been observed that the fates of distinct prophages can be influenced by specific regulatory modules, and that even under identical induction conditions, different combinations of prophage activation can occur [79]. This highlights that, for aquatic pathogens, the relationship between environmental stress and prophage induction is not a single, uniform pathway, but may exhibit considerable prophage-specific and signal-selective features. Moreover, prophage induction should not be equated solely with the SOS pathway. Recent studies indicate that oxidative stress can selectively induce particular prophages through SOS-independent mechanisms [80]. Thus, the RecA–LexA system should be regarded as one important route among several, rather than the sole control mechanism common to all prophages.

From the perspective of pathogen research, heterogeneity of induction within a population is of particular importance. The same environmental stimulus does not necessarily drive all pathogen cells synchronously into the lytic program; some cells may remain in the lysogenic state, whereas others may cross the induction threshold. In Shiga toxin-producing *E. coli* (STEC), RpoS has been implicated in modulating this intercellular heterogeneity, allowing a subset of cells to survive under inducing conditions [81]. Low-frequency spontaneous prophage induction similarly illustrates that, even in the absence of obvious external stimuli, a certain proportion of activated prophage-carrying cells may persist within a pathogen population [82].

In our framework, “decision” does not imply the existence of a universal binary molecular switch within the cell; rather, it refers to the process by which integrated host signals lead a given prophage to cross the induction threshold. Thus, for pathogens, the parameter that may matter more than “whether induction occurs” is how many pathogen cells have crossed the prophage induction threshold, and whether this proportion is sufficient to alter the overall fitness and pathogenic potential of the pathogen population.

### 3.3. How Prophage State Translates into Pathogen Fitness and Virulence-Related Phenotypes

The critical significance of prophages as a “molecular bridge” between the environment and pathogen phenotypes lies in their ability to translate changes in prophage state into functional changes in the host pathogen.

First, when maintained in the lysogenic state, the prophage itself can serve as a genetic functional module. On the one hand, prophages can insert resistance genes directly into the pathogen genome, conferring tolerance to the corresponding antimicrobial agents. On the other hand, prophages frequently carry genes involved in osmoregulation, pH tolerance, heat shock responses, and other environmental stress-related functions, thereby helping pathogens maintain survival under fluctuating external conditions [83]. For example, the gene cargo of Salmonella prophages includes a substantial number of genes involved in carbohydrate and amino acid metabolism, heavy metal and drug resistance, as well as DNA replication and repair regulation, and these genes appear to exert broad effects on host metabolic traits, virulence characteristics, and resistance mechanisms [84].

Second, prophage induction is associated with functional changes in the pathogen. Once a prophage enters the induced state, a subset of pathogen cells undergo lysis; at the same time, this may be accompanied by the release of virulence factors, the production of phage particles, the mobilization of genetic material, and the transfer of pathogenicity-related genes. For example, in *Staphylococcus aureus*, phages released from a subset of cells can kill sensitive competitors and capture beneficial DNA fragments, which are packaged into transducing particles and returned to the prophage-carrying population, thereby driving the transfer of useful traits such as resistance genes [85].

Thus, within a pathogen-oriented framework, the primary output of prophage state changes should not be broadly framed as “ecosystem alteration”; rather, priority should be given to whether the following pathogen-relevant outcomes are affected: enhanced environmental survival, increased stress tolerance, changes in biofilm formation and colonization capacity, alterations in antimicrobial resistance, modulation of virulence factor expression or release, transfer of pathogenicity-related genes, and changes in infectivity.

### 3.4. Prophage Induction: Fitness Benefits Versus Fitness Costs for the Pathogen

Prophage induction carries an inherent duality. The most direct cost is the entry of the host pathogen cell into the lytic cycle and its subsequent death. If a large proportion of pathogen cells are induced synchronously, prophage induction may substantially reduce the pathogen population and may even lead to local population collapse. In addition, induction of a heat-inducible aberrant lambda prophage inserted between the lysA and thyA genes of E. coli has been shown to generate a substantial number of auxotrophic mutants in the surviving cured cell population, suggesting that lambda lysogeny induction may provoke a mutagenic state [86]. However, low-level or localized prophage induction is not necessarily detrimental to the pathogen population as a whole. Lysis of a minority of cells can release phage particles, DNA, and other cellular components, thereby reshaping the competitive environment for the surviving pathogen cells. Studies on spontaneous prophage induction suggest that low-frequency cell lysis can produce population-level consequences that go well beyond the death of individual cells [82]. Thus, from the pathogen’s perspective, the net phenotypic outcome should be evaluated as the sum of adaptive and virulence-related benefits conferred by the prophage, plus the advantages of genetic dissemination and competitive modulation triggered by induction, minus the costs incurred by pathogen cell lysis as well as the genetic and metabolic burdens associated with prophage induction [57,87]. What truly matters here is not prophage induction per se, but whether it ultimately increases or decreases the net fitness of the pathogen in a given environment. When a minor fraction of pathogen cells undergo prophage induction, the bulk of the population can still be maintained, while the phages, genetic material, or virulence-related factors released from the lysed minority may confer indirect benefits on the surviving pathogens [88]. Conversely, when the proportion of induced cells is too high and exceeds the population’s capacity for recovery, the losses resulting from cell death may outweigh any potential benefits, leading to a net decrease in pathogen fitness [88]. For pathogens, this benefit–cost balance is also constrained by clearly defined boundary conditions [89]. For example, prophage-mediated gene transfer and its pathogenic effects depend on the presence of suitable and susceptible recipient bacteria in the environment [90]; the killing of competitors translates into a competitive advantage only when the pathogen and the sensitive bacteria compete for the same ecological niche [91]; and the stress tolerance or resistance genes carried by prophages may enhance pathogen fitness only when the corresponding selective pressure is present [83].

Thus, the same prophage may exhibit different—or even opposite—pathogenic effects depending on the aquaculture conditions.

### 3.5. The Case for a Pathogen-Oriented Multi-Signal Model in Aquaculture

Most mechanistic insights are extrapolated from non-aquatic model systems and require further experimental validation in aquaculture-relevant pathogens. The key distinction between aquaculture systems and traditional single-factor experimental models is that pathogens in aquaculture typically face multiple dynamic environmental pressures simultaneously [92]. Temperature, salinity, dissolved oxygen, nutrient levels, and antimicrobial exposure do not occur in isolation, but rather superimpose across different timescales. For the pathogen, this implies that prophage induction cannot be adequately understood as a one-stimulus, one-response relationship. A more appropriate model is one in which combinations of environmental disturbances alter the integrated physiological state of the pathogen; the prophage responds in a context-specific manner, leading to phenotypic changes in the pathogen that, in turn, translate into shifts in pathogen fitness and pathogenic potential.

First, different environmental signals may carry different weights in their effects on the pathogen. Environmental factors can not only influence prophage status, but also directly affect the growth, metabolism, and virulence regulation of aquatic pathogens. For example, temperature changes can induce extensive transcriptional reprogramming in fish pathogens, and can simultaneously affect both growth and host-colonization-related functions [93]. Thus, an environmental factor that may strongly induce prophages may nonetheless lead to a net decrease in pathogen fitness if it simultaneously severely suppresses pathogen growth. Conversely, an environmental change that induces prophages only weakly but enhances virulence gene expression, biofilm formation, or antimicrobial resistance may have greater pathological significance. Therefore, for aquatic pathogens, the “strongest prophage-inducing signal” does not necessarily equate to the “most important disease risk signal.”

Second, multiple environmental signals may exhibit cross-interactions. Studies have shown that the fate decision of temperate phages can integrate multiple host-sensing pathways simultaneously. For example, in the *Vibrio parahaemolyticus*–VP882 system, the lytic–lysogenic decision of the phage is modulated not only by host quorum-sensing status, but also by whether the bacteria are in a planktonic or surface-attached state, with different host signaling pathways involved depending on the physical environment [94]. This raises the possibility that, in real-world aquaculture settings, multiple inputs—such as hypoxia, nutrient fluctuations, salinity changes, and antimicrobial exposure—may act in concert to alter the metabolic and stress status of the pathogen, resulting in non-additive or even non-linear responses in both prophage induction and its pathogenic outputs.

Third, the temporal order of signals may shape the eventual phenotypic outcome in the pathogen. For example, exposure to nutrient limitation followed by oxidative stress, as opposed to the reverse sequence, may result in different degrees of prophage induction and distinct virulence phenotypes, even when the total exposure levels are identical, owing to differences in the initial physiological state of the pathogen. This environmental history effect is particularly relevant to the periodic or pulsed nature of events in aquaculture, such as hypoxia, feeding, rainfall, and water exchange.

Aquaculture environments also exhibit marked spatial heterogeneity. Pathogens can reside in water, sediment, biofilms, or within the host, and these niches differ considerably in oxygen levels, nutrient availability, cell density, and physiological status [95,96]. Consequently, the same pathogen–prophage combination may display different induction thresholds and pathogenicity-related outputs across distinct ecological niches. From a pathogen-oriented perspective, the significance of this spatial heterogeneity lies not in describing the microbial community as a whole, but in identifying which microenvironment is most conducive to the pathogen gaining advantages in persistence, colonization, antimicrobial resistance, or virulence through its prophages.

## 4. Limitations and Future Directions of Prophage Research in Aquaculture

### 4.1. Limitations

As discussed above, there remains a substantial gap in systematic genomic studies of prophages in aquatic bacterial pathogens, in striking contrast to the mature paradigms established in human and animal research. Although genome annotations exist for specific strains, large-scale, systematic prophage surveys targeting major aquatic pathogens such as *Aeromonas*, *Edwardsiella*, *Flavobacterium*, and *Streptococcus* are lacking; notably, even within the genus *Vibrio*, systematic investigations remain relatively limited [31].

As shown in Table 1, the majority of prophage studies in aquaculture remain at Level 1, that is, limited to computational predictions, with only a limited number having progressed to induction experiments (Level 2). Most existing studies are confined to bioinformatic predictions, and functional validation of the effects of prophage-encoded genes on host phenotypes is extremely scarce. For example, a comparative genomic study identified multiple prophages but merely suggested that they “could be further exploited,” without providing direct experimental evidence—such as changes in virulence, antimicrobial resistance, or environmental adaptability following gene knockout.

Moreover, the accuracy of prediction tools itself is an issue that has received little attention; different tools may yield considerably different predictions for the same genome, yet the vast majority of aquaculture studies rely on a single tool without cross-validation or manual curation. In metagenomic and viromic studies, the identification of prophage sequences also faces serious challenges from contamination and artifacts. Reporting the carriage rates of virulence or resistance genes based solely on “prophage-like contigs” assembled from metagenomic data may substantially overestimate the actual contribution of prophages. Furthermore, most current genomic surveys in aquaculture focus exclusively on “intact prophages” or “prophage-like regions,” while neglecting the presence and functions of defective prophages. Although defective prophages are unable to produce infectious particles, they may still play important roles [55].

In addition, studies on the dynamic changes of prophages across aquaculture cycles remain virtually absent. Although a metagenomic survey of crab aquaculture ponds and ditches revealed associations between prophages and antibiotic resistance genes [50], that investigation was a cross-sectional survey rather than a longitudinal tracking study, and therefore cannot reveal the dynamic evolution of prophages throughout the aquaculture cycle. This gap is particularly significant, because the framework outlined above emphasizes that the environment in which aquatic pathogens reside is not a static backdrop, but rather a dynamic input composed of multiple factors—including temperature, salinity, dissolved oxygen, nutrient status, and antimicrobial exposure. In the absence of time-series data, it would not be possible to test whether prophage induction exhibits threshold effects, or to determine whether the temporal sequence of different environmental stresses alters prophage status and subsequent pathogenic phenotypes in the pathogen.

These deficiencies constitute a major shortcoming in current aquatic disease control research and represent a critical direction for future breakthroughs. Experience from human and animal pathogen research demonstrates that genomic correlations alone are insufficient—only by elucidating the actual contributions of prophage-encoded genes through functional experiments can we truly understand pathogenic mechanisms and resistance dissemination patterns of pathogens and consequently develop precise intervention strategies. Only by addressing these shortcomings can aquatic prophage research advance from “describing correlations” to “elucidating causal mechanisms,” ultimately enabling the understanding, prediction, and intervention of adaptive evolution of pathogens in aquaculture environments.

### 4.2. Strategies and Perspectives

**Figure 3:**
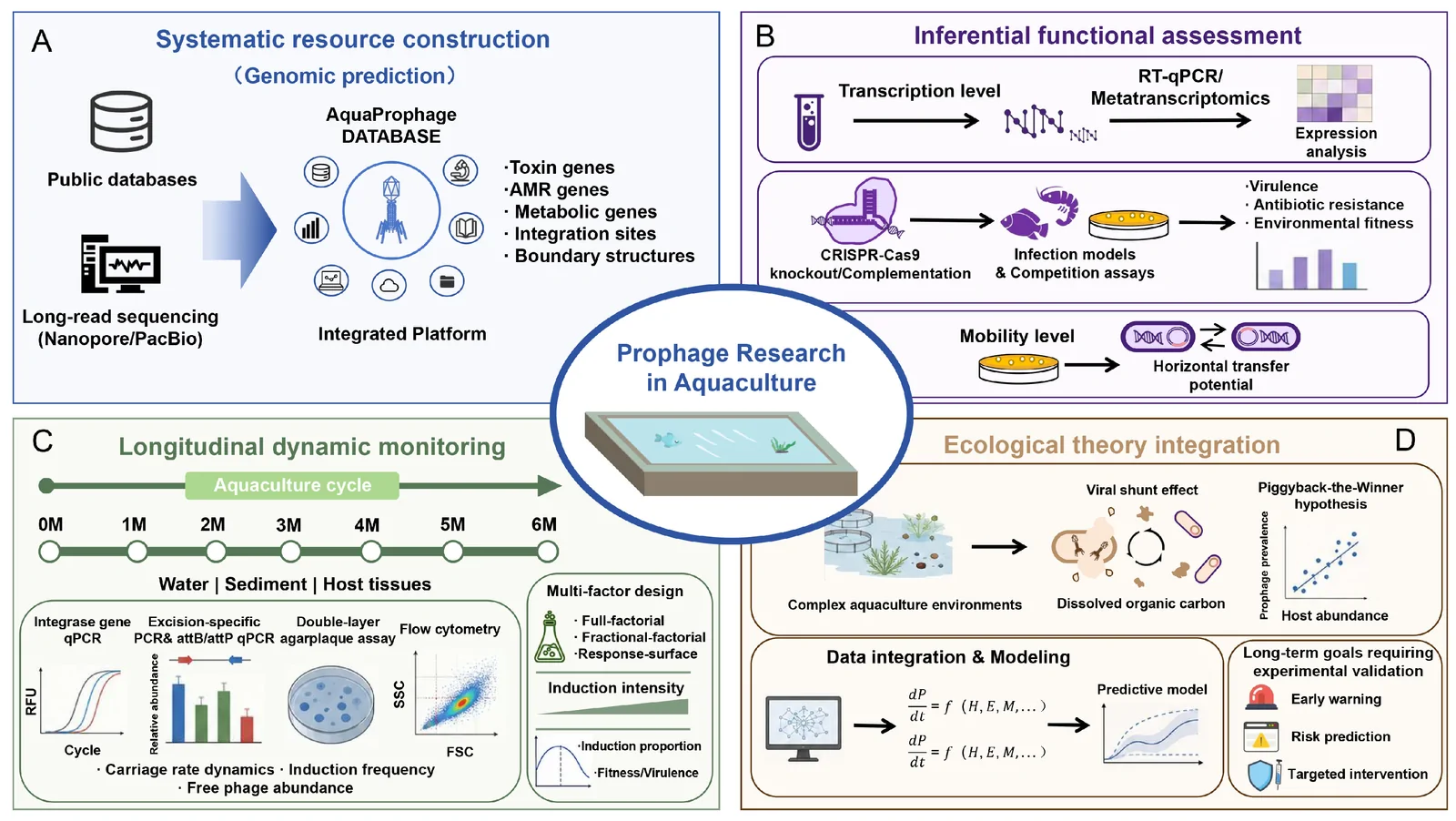
Research roadmap for prophage studies in aquaculture. (**A**) Systematic resource construction based on public databases and long-read sequencing, largely relying on genomic-based computational prediction. (**B**) Inferential functional assessment of prophage-encoded traits. Transcription-level profiling provides expression-supporting evidence; gene knockout/complementation combined with infection-competition assays represents true functional validation; mobility assays indicate predicted horizontal transfer potential rather than confirmed gene-transfer events. (**C**) Longitudinal dynamic monitoring framework for prophage behaviors across aquaculture production cycles. These molecular and phenotypic assays yield indirect evidence for lysogeny and excision-induction events and cannot alone demonstrate horizontal gene transfer. (**D**) Integration of ecological theories and multi-source data modeling. Early-warning, risk-prediction and targeted-intervention applications are presented as long-term goals requiring further field and experimental validation, not currently implementable solutions.

In response to the prominent challenges in current aquaculture prophage research—namely, an abundance of genomic predictions but limited experimental validation, and numerous cross-sectional surveys but few longitudinal studies—we propose the following strategies (Figure 3).

First, we recommend systematically mining publicly available genomic data for the aforementioned pathogens in databases such as NCBI and performing complementary sequencing of representative strains via a hybrid assembly strategy combining long-read (e.g., Nanopore or PacBio) and high-accuracy short-read (e.g., Illumina) sequencing, to accurately resolve prophage integration sites, boundary structures, and genomic arrangements. At the prediction level, multiple tools—including Prophage Hunter, PHASTER, and PhiSpy—should be integrated for parallel identification, with the aim of constructing a dedicated database for prophages in aquatic pathogens (AquaProphage DB) and comprehensively annotating toxin genes, antimicrobial resistance genes, and metabolic-related auxiliary genes [97,98,99,100,101]. The establishment of such a database would help to address the current gap in species coverage and provide a foundational resource for subsequent studies.

Second, functional validation should become a priority direction in the field. Future research should not be limited to bioinformatic predictions; rather, a standardized framework for causal inference should be established. At the transcriptional level, metatranscriptomics or RT-qPCR could be used to screen for the expression activity of prophage genes. At the phenotypic level, gene editing tools such as CRISPR-Cas9 could be employed to construct targeted prophage knockout and complementation mutants [46], with systematic assessment of the actual contributions of prophage-carried genes to host virulence, antimicrobial resistance, and environmental adaptability through infection models (e.g., zebrafish and shrimp) and competitive growth assays. At the mobility level, induction experiments and host-range determination could be used to verify horizontal transfer capacity. In particular, transduction experiments—in which induced phage lysates are co-cultured with recipient strains followed by selection for transductants, combined with PCR verification of stable lysogenization (detection of attB junctions or integration-specific PCR) and phenotypic confirmation of acquired traits (e.g., resistance or virulence)—are essential to conclusively demonstrate prophage-mediated gene transfer. Such an approach would effectively advance the field from “describing correlations” towards “elucidating causal mechanisms”. Notably, successful induction has already been achieved in aquaculture settings [22,32,33,34].

Third, to address the gap in longitudinal dynamic monitoring, we recommend deploying time-series sampling that covers the entire aquaculture cycle (4–6 months), with weekly or bi-weekly collection of water, sediment, and host tissue samples. Critically, it must be recognized that conventional short-read metagenomic time-series can only track fluctuations in prophage sequence abundance and cannot distinguish between increased lysogen abundance and genuine prophage induction events—a distinction that is essential, as an elevated prophage carriage rate may simply reflect proliferation of lysogenic bacteria rather than active induction. To overcome this limitation, a multi-assay strategy should be adopted, with each method serving a distinct interpretive role rather than being used in isolation [102,103,104,105,106,107]. Integrase gene qPCR, when normalized to 16S rRNA or single-copy housekeeping genes (e.g., *rpoB*), provides a high-throughput measure of prophage carriage rate (lysogen abundance), but does not measure induction activity per se. Excision-specific PCR and quantification of attL/attR junctions can indicate the frequency of excision events; a decline in attL/attR abundance relative to the housekeeping gene suggests that a subset of the population has undergone excision, although this signal can also arise from lysogen mortality rather than genuine excision. More definitively, quantification of attB and attP junctions—which are generated only upon successful site-specific recombination during excision—provides a more direct positive indicator of completed excision events than attL/attR alone. Double-layer agar plaque assays quantify infectious phage particles (PFU/mL), yielding functional evidence that excision has progressed through the lytic cycle to produce infectious progeny; by contrast, flow cytometry enumerates total virus-like particles and cannot distinguish infective from non-infective particles. Metagenomic and metatranscriptomic profiling at key time points (e.g., stocking, peak disease period, and harvest) can provide population-level context, capturing both known and unknown prophage diversity and their transcriptional activity. Each method has its own applicable scope and inherent limitations, and their combined use within a tiered framework can effectively compensate for the limitations of any single approach—for example, integrase qPCR serves as a routine screening tool, attB/attP quantification verifies suspected induction events, phage plaque-forming counts confirm functional output, and metagenomics/metatranscriptomics at critical nodes provides background calibration. This longitudinal perspective is essential for revealing prophage dynamics before and after disease outbreaks and for identifying early warning signals of induction. Importantly, these time-series data can be integrated with environmental parameters (temperature, salinity, dissolved oxygen, and antibiotic residues) to identify the specific triggers of prophage induction in aquaculture settings—a critical step towards developing environment-based early warning systems for aquatic disease outbreaks.

It is important to emphasize, however, that integrases are highly diverse, and their abundance alone cannot accurately reflect the induction status or actual activity of prophages in qPCR assays. Moreover, the design of these primers must achieve sufficient specificity to track particular prophages while also possessing sufficient versatility to meet the needs of population-level monitoring. These are practical difficulties that cannot be overlooked and warrant further investigation [108]. Therefore, in actual experimental practice, multi-tiered validation strategies can effectively compensate for the limitations of any single method.

In real-world aquaculture environments, pathogens are rarely, if ever, exposed to a single environmental stress in isolation. Future studies should therefore extend beyond single-factor experiments and incorporate multi-factor designs. Approaches such as full-factorial, fractional-factorial, or response-surface methodologies could be employed to generate combinations of temperature, salinity, hypoxia, nutrient limitation, and antimicrobial exposure, allowing for comparisons between predictions based on single factors and observed outcomes under combined treatments. Such comparisons would help to determine whether the effects of different environmental inputs on prophage induction and pathogen phenotypes are independent, additive, synergistic, or antagonistic. Moreover, disease outbreaks in practice typically occur following a sequence of environmental changes rather than a single instantaneous perturbation, making it particularly important to incorporate temporal order into experimental designs of environmental stress.

Future studies should also examine another prediction raised above: that the impact of prophage induction on the pathogen may depend on the proportion of induced cells, rather than being a binary outcome of “beneficial” versus “detrimental” induction. This could be addressed by manipulating the intensity of environmental stress to establish graded levels of prophage induction, while simultaneously measuring total pathogen abundance, survival rate, recovery rate, biofilm formation, competitive capacity, and infectivity. If, under conditions of low-proportion induction, the bulk of the pathogen population can still be maintained while certain fitness- or virulence-related phenotypes are enhanced, but at high induction proportions both pathogen abundance and infectivity decline markedly, this would suggest that prophage induction may exhibit a non-linear relationship with pathogen fitness.

Beyond these empirical efforts, the findings can be further elevated to the level of systems theory, for instance by integrating marine microbial ecological frameworks. The “Piggyback-the-Winner” hypothesis, which posits that prophage prevalence increases with rising host abundance, can be adapted and extended to aquaculture systems characterized by high host densities and complex environmental conditions [109,110]. The contribution of the “viral shunt” effect to nutrient cycling in aquaculture water bodies [111] can then be quantitatively assessed by coupling with measurements of dissolved organic carbon flux. Embedding molecular-level empirical data into these ecological frameworks ultimately holds the potential to generate predictive and conceptual models for aquaculture, thereby providing a theoretical basis for precision early warning and targeted intervention in aquatic diseases.

In summary, to address the current impasse in aquaculture prophage research—characterized by an abundance of genomic predictions but limited experimental validation, and numerous cross-sectional surveys but few longitudinal studies—coordinated efforts are needed across four interconnected fronts: systematic resource construction, causal mechanism validation, longitudinal dynamic monitoring, and integration with ecological theory.

## 5. Conclusions

In light of the critical appraisal above, the core value of this review lies not in summarizing the known functions of prophages in aquatic pathogens—indeed, direct evidence in this area remains extremely limited—but rather in clearly delineating the mechanistic paradigms established in other fields, so as to provide a source of testable hypotheses for aquaculture research. To this end, we propose a heuristic model, termed the “molecular bridge”, which is intended to integrate existing cross-disciplinary knowledge and generate experimentally verifiable predictions, rather than serving as a simple summary of confirmed mechanisms in aquatic systems. It should be made clear that every step of this framework (from signal input and host perception, through prophage response, to phenotypic output) currently lacks direct experimental support in aquatic pathogens. However, the value of this framework lies precisely in its falsifiability; it offers clear targets for future experimental testing rather than presenting itself as an unchallengeable “truth”.

Furthermore, prophages are not the only mobile genetic elements (MGEs) with ecological and evolutionary significance in bacterial genomes. This review has not systematically compared prophages with other MGEs, such as integrative and conjugative elements (ICEs) or plasmids, and in practice, the synergistic or antagonistic interactions among different elements may be even more complex. Therefore, when reporting that “a given prophage carries gene X”, future studies are advised to simultaneously examine whether the region contains ICE marker genes (for example, tra family genes) and to determine the type of integrase present; distinguishing between the tyrosine recombinase and serine recombinase families is particularly important, so as to more accurately differentiate between prophage-mediated transduction and ICE-mediated conjugation.

Looking ahead, research on prophages in aquatic pathogens urgently needs to shift from “paradigm borrowing” to “local validation” and systematically establish a multi-tiered causal validation system that spans from genomic prediction to functional assays.

## Figures and Tables

**Table 1:** Representative prophage studies in major aquaculture bacterial pathogens.

Bacterial Species and Strain	Host/Source	Prophage Name	Gene	Detection Method	Functional Category	Level of Evidence	Reference
*Edwardsiella anguillarum* EA011113	*Diplodus puntazzo*	Edno5	-	Prophage induction	-	Level 2 *	[22]
*Vibrio parahaemolyticus* A1	*Penaeus vannamei*	-	T6SS	Sequence-based prediction	Virulence-associated genes	Level 1 *	[24]
*Vibrio alginolyticus* B14	*Penaeus vannamei*	-	wbfV/ wcvB, and wecA	Sequence-based prediction	Virulence-associated genes	Level 1 *	[24]
*Aeromonas salmonicid* SRW-OG1	*Epinephelus coioides*	-	-	Sequence-based prediction	-	Level 1 *	[25]
*Aeromonas hydrophila*	Primary fish pathogen	Aeromonas phi 018	-	Sequence-based prediction	-	Level 1 *	[26]
*Aeromonas hydrophila*	Primary fish pathogen	933W, Stx2	-	Sequence-based prediction	Bacterial virulence-related	Level 1 *	[26]
*Flavobacterium succinicans*	Salmonid aquaculture	LMB 10402	-	Sequence-based prediction	-	Level 1 *	[27]
*Flavobacterium psychrophilum*	Salmonid aquaculture	6H	LuxR	Sequence-based prediction	Virulence-associated genes	Level 1 *	[30]
*Streptococcus iniae*	Marine animals	-	-	Sequence-based prediction	-	Level 1 *	[29]
*Vibrio harveyi*	Marine animals	phi354, phiGAN1709	The Flp family of type IV pili	Sequence-based prediction	Virulence-associated genes	Level 1 *	[31]
*Vibrio harveyi*	Marine animals	pAOD13, pGAN1807	ClpP	Sequence-based prediction	Virulence-associated genes	Level 1 *	[31]
*Vibrio harveyi*	Marine animals	phi354	VapD	Sequence-based prediction	Virulence-associated genes	Level 1 *	[31]
*Flavobacterium columnare*	Fish	fF4	-	Prophage induction	-	Level 2 *	[32]
*Vibrio campbellii* HY01	Shrimp	HY01	-	Prophage induction	-	Level 2 *	[33]
*Vibrio campbellii* ATCC BAA-1116	Marine animals	-	-	Prophage induction	-	Level 2 *	[34]

* Level 1, studies based solely on computational predictions without experimental validation; Level 2, studies that have demonstrated inducible prophage activity but lack functional validation of prophage-encoded genes; Level 3, studies that have performed functional validation (e.g., gene knockout, phenotypic assays, or infection models). To date, no aquaculture study has yet reached Level 3.

**Table 2:** Representative paradigms in non-aquatic systems and corresponding evidence in aquatic pathogens.

Translatable Mechanisms	Established Paradigms from Non-Aquatic Systems	Corresponding Evidence in Aquatic Pathogens
Prophage-encoded toxins	CTXφ–cholera toxin in *V. cholerae*; Shiga toxin in STEC; diphtheria toxin in *C. diphtheriae*	Stx2 prophages in *A. hydrophila* strains isolated from fish
Remodeling of cell surface and host interactions	Cell wall alterations in *S. marcescens*; surface property changes and increased EPS production in *R. solanacearum*	phi354 and phiGAN1709 encode Flp-related genes; a Mu-like prophage in *V. parahaemolyticus* CHN25 affects motility, biofilm, and cytotoxicity
Direct mediation of bacterial infectivity by prophages	Gifsy-2 gtgE/sodCI promote *Salmonella* survival during infection	T6SS-related genes in *V. parahaemolyticus*; ATP-dependent Clp protease in pAOD131/pGAN1807; VapD in phi354; PadR in *V. harveyi* phages
Prophage as a regulatory switch	*L. monocytogenes* prophage activates upon macrophage infection, promoting intracellular growth and virulence	-

## Data Availability

No new data were created or analyzed in this study. Data sharing is not applicable to this article.
